# Seasonal Variation of Antiretroviral Drug Exposure during the Year: The Experience of 10 Years of Therapeutic Drug Monitoring

**DOI:** 10.3390/biomedicines9091202

**Published:** 2021-09-12

**Authors:** Jessica Cusato, Jacopo Mula, Alice Palermiti, Alessandra Manca, Miriam Antonucci, Valeria Avataneo, Elisa Delia De Vivo, Alice Ianniello, Andrea Calcagno, Giovanni Di Perri, Amedeo De Nicolò, Antonio D’Avolio

**Affiliations:** 1 Department of Medical Sciences, Laboratory of Clinical Pharmacology and Pharmacogenetics, University of Turin, Amedeo di Savoia Hospital, 10149 Turin, Italy; jessica.cusato@unito.it (J.C.); jacopo.mula@unito.it (J.M.); miriam.antonucci20@gmail.com (M.A.); valeria.avataneo@unito.it (V.A.); elisa.devivo59@gmail.com (E.D.D.V.); alice.ianniello89@gmail.com (A.I.); amedeo.denicolo@unito.it (A.D.N.); antonio.davolio@unito.it (A.D.); 2 Department of Medical Sciences, Unit of Infectious Diseases, University of Turin, Amedeo di Savoia Hospital, 10149 Turin, Italy; andrea.calcagno@unito.it (A.C.); giovanni.diperri@unito.it (G.D.P.)

**Keywords:** cytochromes, plasma exposure, pharmacokinetics, seasonality, gene expression

## Abstract

Although studies show an annual trend for immunosuppressive drugs, particularly during different seasons, no data are available for antiretroviral drugs exposures in different periods of the year. For this reason, the aim of this study was to investigate an association between seasonality and antiretroviral drugs plasma concentrations. Antiretroviral drugs exposures were measured with liquid chromatography validated methods. A total of 4148 human samples were analysed. Lopinavir, etravirine and maraviroc levels showed seasonal fluctuation. In detail, maraviroc and etravirine concentrations decreased further in summer than in winter. In contrast, lopinavir concentrations had an opposite trend, increasing more in summer than in winter. The etravirine efficacy cut-off value of 300 ng/mL seems to be affected by seasonality: 77.1% and 22.9% of samples achieved this therapeutic target, respectively, in winter and summer, whereas 30% in winter and 70% in summer did not reach this value. Finally, age over 50 years and summer remained in the final multivariate regression model as predictors of the etravirine efficacy cut-off. This study highlights the seasonal variation in antiretroviral drugs plasma concentrations during the year, leading to a better understanding of inter-individual variability in drug exposures. Studies are required in order to confirm these data, clarifying which aspects may be involved.

## 1. Introduction

Seasonality has been documented in many biological markers such as hormones; in fact, an annual fluctuation was highlighted for different environmental factors such as infections, ultraviolet (UV) exposure and other factors such as vitamin D (VD) [1]. Particularly, some studies in the literature have illustrated correlations between clinical conditions or drug plasma levels and seasonality. In this context, Bauer et al. described the seasonal variability of asthma exacerbation in patients suffering from uncontrolled pathology, monitoring lung functions metrics and asthma symptoms. The authors highlighted a progressive worsening of uncontrolled asthma towards winter, unlike surges in asthma exacerbations during spring and autumn periods [2].

Concerning inflammatory bowel disease (IBD), season of birth seems to be associated with a higher or lower risk of developing this autoimmune disease (AD), considering the variation of VD levels in different seasons. The majority of IBD patients were born in winter (particularly in January and February); in contrast, a nadir for IBD positivity was registered in autumn [3].

Specifically, considering HIV and AIDS, some studies showed a seasonal pattern [4,5]. For example, a study performed in Africa evidenced a seasonal AIDS distribution, with outbreaks in particular geographic areas in the first eight months of the year [6]. In this context, it is important to highlight that a possible relationship between AIDS annual spreading and seasonal antiretroviral drug (ARV) concentrations have not been proposed yet. In fact, in this field, the study of Lindh et al. showed that two immunosuppressive drugs, tacrolimus and sirolimus, have an annual variation in concentrations; specifically, in spring/summer, their levels are lower than in autumn/winter [7]. Drug fluctuation seems to show an opposite trend compared to levels of VD, which modulates the expression of genes encoding cytochromes (CYPs, e.g., CYP3A5) and transporters (e.g., ABCB1), involved in tacrolimus and sirolimus metabolism and transport [8]. In fact, in vitro studies indicate that VD induces CYPs and drug transporter gene expression through its receptor (VDR)-mediated increase in transcription; this activity results in higher CYPs drug substrates metabolism and transport [9,10].

Considering that immunosuppressive drugs such as ARVs are administered for a long period (all lifelong), studies have to clarify a possible role of seasonality in affecting ARV concentrations.

For this reason, the aim of this study was to evaluate the anti-HIV drug plasma level trend during the year through a therapeutic drug monitoring (TDM) repository in order to understand if they could have a seasonal variation, similar to what has been suggested for immunosuppressive drugs.

Furthermore, it was assessed whether the season, in addition to other patient-related factors, will be able to predict the concentration cut-offs associated with ARV efficacy or toxicity.

## 2. Materials and Methods

### 2.1. Study Design

TDM is a clinical practice able to quantify drugs in different biological matrices, particularly in plasma, leading to dose optimisation in order to achieve efficacious treatment, avoiding toxicity. A TDM record of 10 years was analysed: samples collected from people living with HIV (PLWH) treated at the Amedeo di Savoia (Turin, Italy) were evaluated. Samples of patients with age ≥ 18 years, good general condition (without other diseases), on ARV therapy for >7 days, absence of any interacting drugs (such as rifampicin, methadone or erythromycin), no co-infection, drug intake before blood withdrawal and reported medication adherence above 90% were considered (Ethics Committee approvals: CS2/325 del 8/8/2017).

For each patient, the following data were provided in the register: demographics (sex, age), concomitant medications at the time of the visit, antiretroviral therapy in progress and time and date of the last administration of ARVs.

### 2.2. ARV Plasma Concentrations

Samples were selected considering patients who were not supplemented with VD.

Sampling was performed at a steady state before drug dose administration (Ctrough). Plasma samples were obtained from a lithium–heparin tube (7 mL) and were stored in cryovials at −20 °C before analysis. The following drugs were quantified: etravirine (ETV), maraviroc (MVC), lopinavir (LPV), darunavir, atazanavir, ritonavir, raltegravir, dolutegravir, abacavir, tenofovir, nevirapine, lamivudine and emtricitabine.

Drug concentrations were determined by ultra/high-performance liquid chromatography (UPLC/HPLC), according to previously described and fully validated methods [11,12,13]. Samples with undetectable concentrations were considered non-adherent and were excluded from the analyses.

Therapeutic ranges for analyzed drugs were considered in accordance with those reported by Pretorius et al. [14].

### 2.3. Statistical Analysis

All the continuous variables were tested for normality with the Shapiro–Wilk test. The correspondence of each parameter was evaluated with a normal or non-normal distribution through the Kolmogorov–Smirnov test. Non-normal variables were described as median values and interquartile range (IQR), and categorical variables as numbers and percentages. Kruskal–Wallis and Mann–Whitney tests were adopted for differences in continuous variables between seasons, considering a statistical significance with a two-sided *p*-value < 0.05.

Stepwise multivariate logistic regression analyses were performed to predict drug cut-off values (ETV).

All tests were performed with IBM SPSS Statistics for Windows v.26.0 (IBM Corp., Chicago, IL, USA).

## 3. Results

In this study, 4148 samples were considered; their characteristics are provided in Table 1. Percentages of treatment combinations are summarised in Table S1.

**Figure 1:**
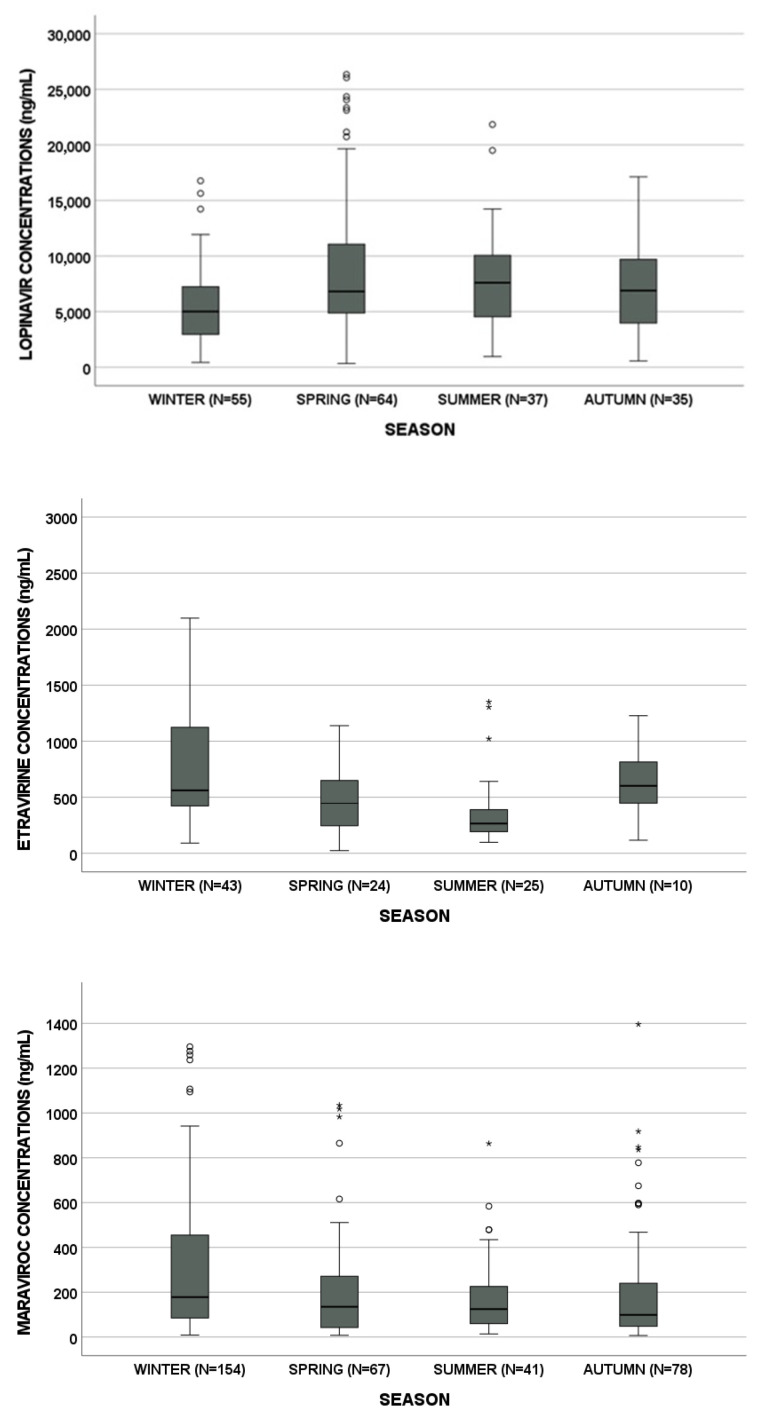
Lopinavir, etravirine and maraviroc concentrations according to seasonal variation. Circles and stars indicate “out” values (small circle) and “far out” values (star).

**Figure 2:**
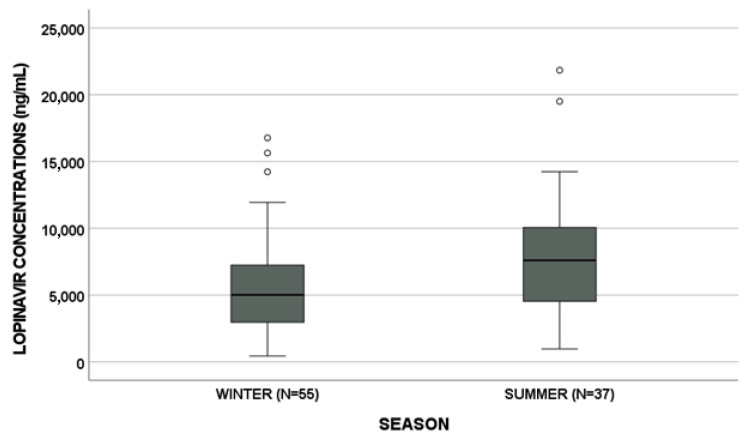

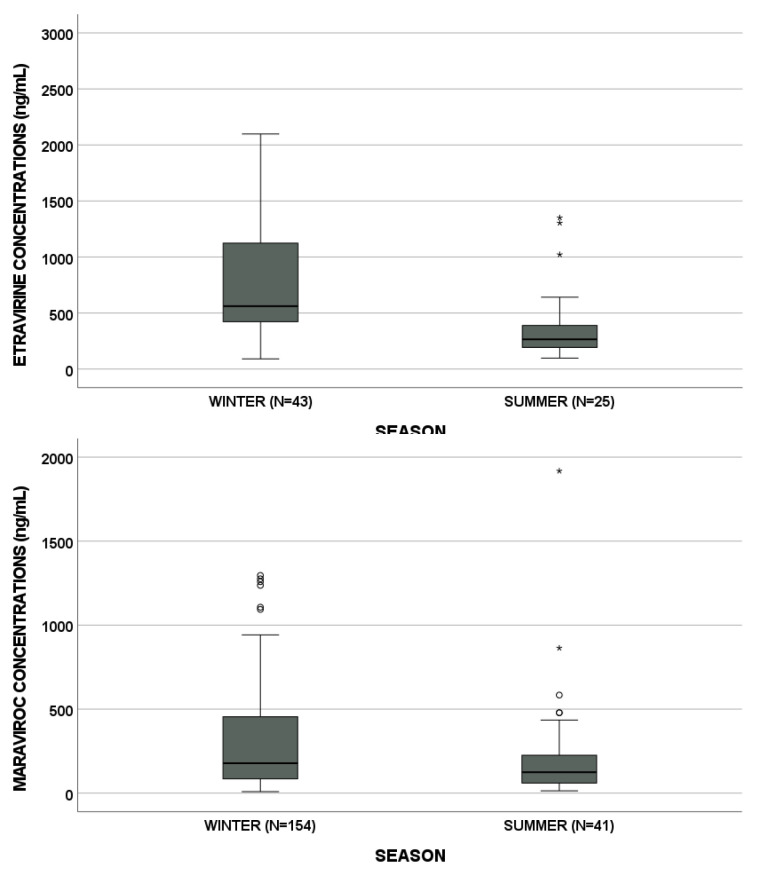
Lopinavir, etravirine and maraviroc levels in winter vs. summer. Circles and stars indicate “out” values (small circle) and “far out” values (star).

LPV, ETV and MVC concentrations showed a seasonal trend (*p* = 0.006, *p* = 0.002 and *p* = 0.001, respectively, Figure 1). In detail, LPV, ETV and MVC median concentrations are reported in Table 2. Particularly, MVC and ETV concentrations were lower in summer than in winter (*p* = 0.033 and *p* < 0.001, respectively). On the other hand, LPV concentrations showed an opposite trend (*p* = 0.003, Figure 2).

Furthermore, our other aim was to evaluate if seasonality could have a role in affecting the achievement of concentrations associated with drug efficacy or toxicity. ETV was the only ARV showing statistical significance (*p* < 0.001) considering its therapeutic efficacy cut-off value of 300 ng/mL.

**Figure 3:**
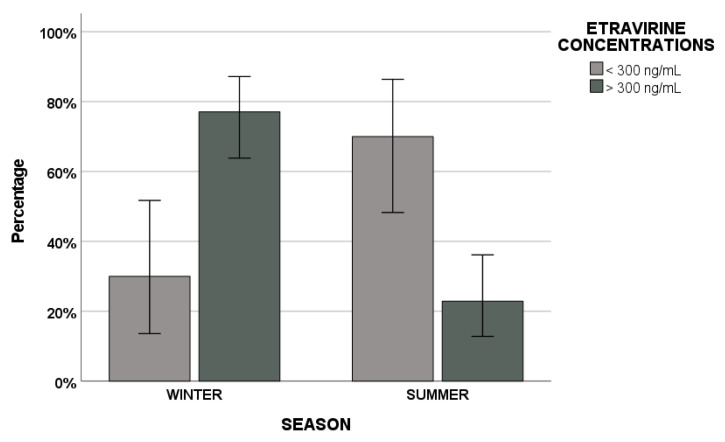
Etravirine efficacy concentration cut-off value of 300 ng/mL in summer vs. winter.

Plasma samples with an ETV higher than 300 ng/mL were 37 (77.1%) in winter but 11 (22.9%) in summer; samples with concentrations lower than this cut-off were 6 (30%) in winter but 14 (70%) in summer (Figure 3).

In addition, a statistically significant difference between the percentage of samples with ETV levels higher and lower than 300 ng/mL was highlighted both in winter and summer (*p* < 0.001 and *p* < 0.001).

**Figure 4:**
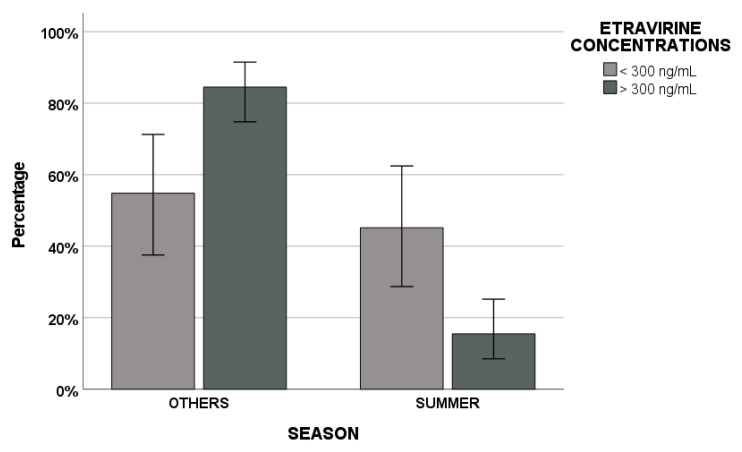
Etravirine efficacy concentration cut-off value of 300 ng/mL in summer vs. other seasons.

Finally, different patients demographic, seasonal and pharmacological elements were evaluated as predictors of ETV effectiveness cut-off value through regression analysis (Table 3). Age ≥ 50 years, summer, winter and proton-pump inhibitor (PPI) co-administration were retained in the univariate model, whereas age over 50 years and summer remained in the final multivariate regression model (Figure 4).

No difference in ARV concentrations was suggested for monthly evaluation or gender.

## 4. Discussion

Since HIV medications, similar to immunosuppressive drugs, are administered for a long time (lifetime), we aimed to investigate if seasonality could have a role in affecting ARV concentrations. In this context, TDM serves as a potent tool to monitor drug concentrations in order to understand if they are within the therapeutic range or, for example, to clarify patient compliance.

Supporting this, our group recently submitted (data not published) a study aimed to analyse the annual trend of efavirenz (EFV) exposure. As suggested for tacrolimus and sirolimus, also for EFV, seasonality could have an impact in terms of its plasma variation [15].

In this study, 4148 HIV drug quantifications reported in a 10-year TDM experience were considered: LPV exposure was higher in spring and summer and lower in autumn and winter; in contrast, ETV and MVC had an opposite trend.

Moreover, we evaluated the association between seasonality and the achievement of cut-off plasma concentrations related to efficacy or toxicity. ETV was the only ARV to reach statistical significance, considering its cut-off of 300 ng/mL associated with therapeutic effectiveness. In winter, the percentage of samples reaching 300 ng/mL was higher compared with non-achieving ones and vice versa in summer.

Finally, in this study, factors able to predict this ETV cut-off value were evaluated through regression analysis. While age ≥ 50 years, summer, winter and PPI concomitant drugs remained in the univariate regression analysis, age ≥ 50 years and summer were retained in the final multivariate regression model. Particularly, the percentage of samples with ETV > 300 ng/mL was lower in summer compared with other seasons. According to what has been shown by Lindh et al., we could suppose a possible VD contribution, but data have to be analysed in further studies. In fact, they showed that tacrolimus and sirolimus immunosuppressive agent concentrations decreased with increased VD levels; this could be due to a VD inductive effect on genes encoding for proteins involved in these drugs’ metabolism and excretion (CYP3A5, CYP2B6 and ABCB1 genes encoding for CYP3A5, CYP2B6 enzymes and for P-glycoprotein transporter, respectively) [7,8].

One limitation of this study is that a small number of samples were taken into consideration monthly; in fact, no difference in ARV plasma exposures during the year was evidenced according to months. Consequently, a larger number of patients have to be enrolled monthly in future. In addition, another limitation is that water consumption is deeply influenced by the seasons; thus, the levels of different molecules (such as drugs) could vary. Further studies focused on this aspect have to be performed.

Finally, VD levels could be quantified in order to understand if they could impact anti-HIV concentrations annual fluctuations.

## 5. Conclusions

In conclusion, this is the first study reporting the seasonal variation in ARV plasma exposures in a cohort of PLWH in ten years, particularly for LPV, ETV and MVC. This study could be useful to achieve a better explanation of inter-individual variability in drug exposures, leading to superior management of patient treatment. In future, works are required in order to better clarify this aspect.

## Figures and Tables

**Table 1:** Characteristics of the described population.

Characteristics	
Number of patients (*n*)	4148
Age (years), median (range interquartile [IQR])	52 (47–55)
Male sex, *n* (%) Female sex, *n* (%)	2846 (69%) 1302 (31%)
Weight, median [IQR]	69 [58–78]
Height, median [IQR]	170 [164–176]
Concomitant drugs, *n* (%)	1572 (37.9%)
Proton-pump inhibitors, *n* (%)	225 (5.4%)
Patients treated with nevirapine, *n* (%)	165 (4%)
Patients treated with raltegravir, *n* (%)	518 (12.5%)
Patients treated with darunavir, *n* (%)	412 (9.9%)
Patients treated with ritonavir, *n* (%)	936 (22.6%)
Patients treated with atazanavir, *n* (%)	721 (17.4%)
Patients treated with etravirine, *n* (%)	102 (2.5%)
Patients treated with abacavir, *n* (%)	137 (3.3%)
Patients treated with tenofovir, *n* (%)	1612 (38.9%)
Patients treated with emtricitabine, *n* (%)	1547 (37.3%)
Patients treated with maraviroc, *n* (%)	340 (8.2%)
Patients treated with lopinavir, *n* (%)	191 (4.6%)

**Table 2:** Lopinavir, etravirine and maraviroc median concentrations (ng/mL) in relation to seasonality.

Season	Lopinavir Median Concentrations (ng/mL) [IQR]	Etravirine Median Concentrations (ng/mL) [IQR]	Maraviroc Median Concentrations (ng/mL) [IQR]
Winter	5015 [2009–7541]	562 [410–1133]	178.5 [84.5–456.5]
Spring	6829 [4839–11148.5]	447 [234.5–660]	135 [42–273]
Summer	7608 [4396–1012.5]	265 [179–448.5]	125 [57–259.5]
Autumn	6906 [3678–10312]	602 [372.25–854]	99 [46.75–240.25]

**Table 3:** Logistic regression analysis for predictors of etravirine effectiveness cut-off value. In bold, statistically significant values.

Etravirine Efficacy Cut-Off Value of 300 ng/mL				
	Univariate Regression		Logistic Regression	
Predictive Factors	*p*-Value	OR (95% IC)	*p*-Value	OR (95% IC)
Age ≥ 50	**0.037**	**2.5 (1.1–6.0)**	**0.006**	**4.6 (1.5–13.8)**
Gender	0.564	1.3 (0.5–3.3)		
Weight ≥ 70 kg	0.476	0.7 (0.3–1.7)		
Height ≥ 170 cm	0.091	0.5 (0.2–1.1)		
Summer	**0.002**	**0.2 (0.1–0.6)**	**0.017**	**0.2 (0.1–0.8)**
Winter	**0.003**	**4.5 (1.7–12.4)**	0.077	2.9 (0.9–9.3)
Proton-pump inhibitors	**0.006**	**0.3 (0.1–0.7)**	0.188	0.5 (0.2–1.4)

## Data Availability

Data are available on request by the corresponding author.

## Supplementary Materials

The following are available online at https://www.mdpi.com/article/10.3390/biomedicines9091202/s1, Table S1: Combination percentage of antiretroviral treatment.
