# Supplementary material for: Seasonal Variation of Antiretroviral Drug Exposure during the Year: The Experience of 10 Years of Therapeutic Drug Monitoring
Source: Biomedicines. 2021 Sep 12;9(9):1202. doi: 10.3390/biomedicines9091202 (PMC8468337; doi:10.3390/biomedicines9091202)
Supplement: Supplementary file 1 [file biomedicines-09-01202-s001.zip › biomedicines-1375829-supplementary.pdf]

[%]	Nevirapine	Atazanavir	Lopinavir	Abacavir	Tenofovir	Maraviroc	Raltegravir	Emtricitabine	Etravirine	Ritonavir	Efavirenz	Darunavir
**Nevirapine**	/	-	0,04	0,5	1,3	0,1	1,6	1,3	-	0,1	-	-
**Atazanavir**	-	/	-	1,2	12	1	2,4	11,6	0,04	9,6	-	-
**Lopinavir**	0,04	-	/	0,2	2,6	0,3	0,9	2,4	0,4	4,1	-	-
**Abacavir**	0,5	1,2	0,2	/	-	0,3	0,5	-	-	1,1	0,1	0,3
**Tenofovir**	1,3	12	2,6	-	/	2,5	4,6	36,8	0,7	14,2	7,4	4,2
**Maraviroc**	0,1	1	0,3	0,3	2,5	/	1,8	2	0,4	4,6	-	4
**Raltegravir**	1,6	2,4	0,9	0,5	4,6	1,8	/	4,1	1,3	4,6	0,04	2,7
**Emtricitabine**	1,3	11,6	2,4	-	36,8	2	4,1	/	0,6	13,4	7,4	3,7
**Etravirine**	-	0,04	0,4	-	0,7	0,4	1,3	0,6	/	1,3	-	0,8
**Ritonavir**	0,1	9,6	4,1	1,1	14,2	4,6	4,6	13,4	1,3	/	0,02	8,5
**Efavirenz**	-	-	-	0,1	7,4	-	0,04	7,4	-	0,02	/	0,02
**Darunavir**	-	-	-	0,3	4,2	4	2,7	3,7	0,8	8,5	0,02	/

Supplementary Table S1 :Combination Percentage of antiretroviral treatment
